# Editorial: Tumor ablation and immunity

**DOI:** 10.3389/fimmu.2023.1291918

**Published:** 2024-02-22

**Authors:** Ming Kuang

**Affiliations:** Department of Liver Surgery, Center of Hepato-pancreato-biliary Surgery, The First Affiliated Hospital of Sun Yat-sen University, Guangzhou, China

**Keywords:** tumor, ablation, immunity, combination (combined) therapy, immunotherapy

Tumor ablation techniques, which involve the localized destruction of tumors using heat, cold, or chemistry materials et al, have long been utilized as curative or palliative treatments for various cancer types, such as liver cancer, breast cancer, lung cancer, and so on. Ablation caused cell deaths could effectively exposes tumor antigen and triggers an anti-tumor immune response. The intersection of tumor ablation and immunity has emerged as a promising frontier in cancer therapy. In this editorial, we delve into the significance of this evolving field by examining several key researches, highlighting their contributions to our understanding of tumor ablation and immunity, and shedding light on the prospects of transforming cancer care.

## Elevating antitumor immunity through ablation

The Study by Peng et al. exemplifies the symbiotic relationship between tumor ablation and immunity. In this study, Peng et al. present a novel tumor ablation approach known as cryo-thermal therapy. This technique, which combines freezing and heating to ablate tumors locally, not only achieves impressive rates of tumor eradication but also triggers a profound anti-tumor immune response. Cryo-thermal therapy was found to induce Th1-dominant CD4+ T cells, a specific type of immune cell that plays a crucial role in systematic antitumor immune memory. These activated CD4+ T cells were shown to directly inhibit tumor growth, enhance the activity of cytotoxic immune cells, and promote the maturation of antigen-presenting cells, all of which collectively contribute to a potent antitumor immune response.

Another study by Lou et al. also revealed the potential of tumor ablation to enhance antitumor immunity. This research focuses on myeloid-derived suppressive cells (MDSCs), a critical challenge in cancer therapy, which are considered to be suppressive and inhibit antitumor immunity. By combining cryo-thermal therapy with all-trans retinoic acid (ATRA), the study succeeded in promoting the conversion of MDSCs into potent antigen-presenting cells, shifting the immune landscape in favor of antitumor responses. This combination therapy not only disrupted the suppressive immune environment but also led to improved survival rates in preclinical models of breast cancer.

## Decoding immune shifts for outcome prognosis

Decoding the immune microenvironment shifts after ablation is crucial for identifying novel biomarkers for prognosis as well as for patients selection of immunotherapy. The study from Zhang et al. examined the immune cell subsets and cytokine profiles in the peripheral blood of patients with pulmonary malignancies before and after microwave ablation. They observed a significant increase in the proportion of CD8+ T cells, while a decrease in regulatory T cells (Tregs). This reduction in Tregs correlated with improved progression-free survival, highlighting the potential of immune cell dynamics as prognostic indicators.

Zang et al. 's study delves into the complex relationship between immune dynamics and cancer recurrence after thermal ablation. The study examined tumor-associated antigen (TAA) specific T-cell responses in patients with hepatocellular carcinoma (HCC) before and after thermal ablation. Interestingly, the researchers observed a shift in T-cell responses from AFP-specific to SMNMS-specific following ablation. This shift was associated with improved survival outcomes. However, the immune response appeared to be transient, as antitumor immune response improved significantly at one-week, but the T cells exhibited a more exhausted phenotype at later time points (4-weeks post ablation). The study highlights the intricate nature of the immune response post-ablation and the ablation-induced anti-tumor immunity. These results might have the potential to inform combination strategies aimed at sustaining long-term immune-mediated tumor control.

## Revolutionizing treatment strategies by combining ablation and immunotherapy

Moving from basic research to clinical application, Li et al. exemplifies the translation of novel approaches into the clinical scenario. This study combined microwave ablation with targeted therapy (apatinib) and immunotherapy (camrelizumab) in 14 patients with advanced hepatocellular carcinoma. The combination demonstrated impressive clinical activity, resulting in overall response rate (ORR) of 50.0% and 10.8 months and 19.3 months, respectively.

Shi et al. demonstrated that the combination of microwave ablation, TACE, and PD-1 inhibitor might be a favorable treatment option for HCC who are intolerant to TKIs. The combination therapy resulted in better PFS (10.0 vs. 4.7 months) and OS (17.0 vs. 8.5 months) compared to microwave ablation plus TACE. The treatment method and Child-Pugh class were identified as independent prognostic factors. While adverse events were reported, they were similar to the microwave ablation plus TACE group, suggesting the potential clinical benefit of adding PD-1 inhibitor to microwave ablation and TACE.

Another pilot study including 9 patients by Wei et al. demonstrated a promising objective response rate of 22.22% in patients with advanced solid tumors who received cryoablation and intra-arterial PD-1 inhibitors after failing previous checkpoint inhibitor therapy. The combination therapy was associated with macrophage polarization and an increase in circulating CD4+ T cells. While a majority of patients experienced progressive disease, the therapy showed potential efficacy and controllable safety for overcoming immune resistance in advanced solid cancers.

## New era in cancer care

The studies featured in this editorial collectively underscore the transformative potential of decoding the interaction of tumor ablation and immunity, from inducing anti-tumor immunity and predicting outcomes to designing combined treatment regimens. As researchers and clinicians continue to explore this evolving landscape, collaboration and translational studies will be instrumental in utilizing the synergetic potential of ablation and immunotherapy, leading to a new era in cancer therapy.

## Author contributions

MK: Conceptualization, Writing – original draft, Writing – review & editing.

